# Knockout of endothelin type B receptor signaling attenuates bleomycin-induced skin sclerosis in mice

**DOI:** 10.1186/s13075-016-1011-4

**Published:** 2016-05-21

**Authors:** Kengo Akashi, Jun Saegusa, Sho Sendo, Keisuke Nishimura, Takuya Okano, Keiko Yagi, Masashi Yanagisawa, Noriaki Emoto, Akio Morinobu

**Affiliations:** Department of Rheumatology and Clinical Immunology, Kobe University Graduate School of Medicine, 7-5-1, Kusunoki-cho, Chuo-ku, Kobe, 650-0017 Japan; Department of Clinical Pharmacy, Kobe Pharmaceutical University, Kobe, Japan; Division of Cardiovascular Medicine, Department of Internal Medicine, Kobe University Graduate School of Medicine, Kobe, Japan; International Institute for Integrative Sleep Medicine (WPI-IIIS), University of Tsukuba, Tsukuba, Japan

**Keywords:** Systemic sclerosis, Endothelin type B receptor, Dermal fibroblast

## Abstract

**Background:**

Endothelin-1 (ET-1) is important in the pathogenesis of systemic sclerosis (SSc). ET-1 binds two receptors, endothelin type A (ET_A_) and endothelin type B (ET_B_). Dual ET_A_/ET_B_ receptor antagonists and a selective ET_A_ receptor antagonist are used clinically to treat SSc, and the effect of these antagonists on fibroblast activation has been described. However, the role of ET_B_ receptor signaling in fibrogenesis is less clear. This study was conducted to evaluate the profibrotic function of ET_B_ receptor signaling in a murine model of bleomycin (BLM)-induced scleroderma.

**Methods:**

We used ET_B_ receptor–knockout (ET_B_KO) mice, which are genetically rescued from lethal intestinal aganglionosis by an ET_B_ receptor transgene driven by the human dopamine β-hydroxylase (DβH)-gene promoter, and wild-type mice with DβH-ET_B_ (WT). BLM or phosphate-buffered saline (PBS) was administered subcutaneously by osmotic minipump, and skin fibrosis was assessed by dermal thickness, subcutaneous fat atrophy, and myofibroblast count in the dermis. Dermal fibroblasts isolated from ET_B_KO and WT mice were cultured in vitro, stimulated with BLM or ET-1, and the expression of profibrotic genes was compared by quantitative PCR.

**Results:**

Dermal thickness, subcutaneous fat atrophy, and myofibroblast counts in the dermis were significantly reduced in ET_B_KO mice compared to WT mice, after BLM treatment. Compared with wild-type, dermal fibroblasts isolated from ET_B_KO mice showed lower gene expressions of α-smooth muscle actin and collagen 1α1 in response to BLM or ET-1 stimulation in vitro*.*

**Conclusions:**

ET-1–ET_B_ receptor signaling is involved in skin sclerosis and in collagen synthesis by dermal fibroblasts.

**Electronic supplementary material:**

The online version of this article (doi:10.1186/s13075-016-1011-4) contains supplementary material, which is available to authorized users.

## Background

Systemic sclerosis (SSc), a connective-tissue disease of unknown etiology, is characterized by autoimmunity, microvascular impairment, chronic inflammation, and fibrotic changes in the skin and internal organs [1]. The pathogenesis of SSc has been studied in a bleomycin (BLM)-induced scleroderma model [2, 3]. Among fibrotic agents known to contribute to scleroderma, the role of endothelin-1 (ET-1) in SSc fibrosis has been well described [4]. The plasma concentration of ET-1 is elevated in SSc patients, and endothelin receptor expression is increased in the lungs and skin [5, 6]. ET-1 plays an important role in murine model of BLM-induced scleroderma because endothelin receptor antagonist therapy is reported to attenuate skin fibrosis of mice [4]. Recently, vascular endothelial cell-specific ET-1 knockout mice are also reported to attenuate skin sclerosis [7]. ET-1, which was first characterized as a potent vasoconstrictive peptide derived from endothelial cells, is an important profibrotic mediator that induces the differentiation of fibroblasts to myofibroblasts and increases the extracellular matrix. Therefore, ET-1 plays a critical role in vascular impairment and fibrosis in SSc patients.

ET-1 binds two receptor subtypes, the endothelin type A (ET_A_) and endothelin type B (ET_B_) receptors [8]. Treatment with a dual ET_A_/ET_B_ receptor antagonist or a selective ET_A_ receptor antagonist improves mortality in patients with pulmonary arterial hypertension [9–11]. ET_A_ receptor signaling is critical for dermal fibroblast activation [12]. However, the role of ET_B_ receptor signaling in fibrogenesis is less clear.

To investigate the profibrotic activity of the ET-1–ET_B_ receptor signal in fibrosis, we used endogenous ET_B_ receptor–knockout (ET_B_KO) mice with an ET_B_ receptor transgene driven by the human dopamine β-hydroxylase (DβH) gene promoter, which generated “rescued” ET_B_-knockout mice [13–15]. ET_B_-receptor mutant mice and general (not rescued) ET_B_KO mice, which have aganglionic megacolon and white spotting on the coat, die before reaching adulthood because ET_B_ receptor signaling is important in generating melanocytes and enteric neurons [16]. In the “rescued” ET_B_KO mice, a functional ET_B_ receptor is expressed only in the enteric nervous system; other tissues lack ET_B_ receptors. These mice survive into adulthood [17].

Here we show that ET_B_KO mice were resistant to BLM-induced scleroderma, and that compared to WT mice, ET_B_KO mice showed less fibroblast activation and myofibroblast formation in response to BLM or ET-1.

## Methods

### Animals

The mice were housed in the animal facility of Kobe Pharmaceutical University, with a 12-h dark/light cycle and ad libitum water and food. Heterozygous ET_B_KO mice (ET_B_+/-) on a C57BL/6 J genetic background were generated as described previously [17, 18]. The human DβH gene promoter–regulated ET_B_ receptor transgene (DβH-ET_B_) and ET_B_+/- with DβH-ET_B_ were generated as described previously [13–15]. ET_B_+/- mice were crossed with DβH-ET_B_ mice to obtain three genotypes: wild-type ET_B_+/+ with DβH-ET_B_ (WT mice), ET_B_+/- with DβH-ET_B_ (heterozygous KO mice), and ET_B_-/- with DβH-ET_B_ (ET_B_KO mice). The ET_B_KO mice had a partly white coat that distinguished them from heterozygous KO and WT mice. All experimental protocols were approved by the Ethics Review Committee for Animal Experimentation of Kobe Pharmaceutical University.

### Reagents and antibodies

BLM and ET-1 (H-6995) were purchased from Nippon-Kayaku (Tokyo, Japan) and Bachem (Bubendorf, Switzerland), respectively. Dulbecco's modified Eagle's medium (DMEM) (Nissui-Seiyaku, Tokyo, Japan), RPMI 1640 (Wako Pure Chemical Industries, Osaka, Japan), fetal bovine serum (FBS) (MP Biomedicals, Santa Ana, CA, USA), 1 % penicillin–streptomycin (Lonza, Basel, Switzerland), L-glutamine (Thermo Fisher Scientific, Waltham, MA, USA), and type 1 collagenase (Worthington Biochemical Corporation, Lakewood, NJ, USA) were also used. Bovine serum albumin (BSA), 2 M acetic acid, and dispase were purchased from Sigma-Aldrich (St. Louis, MO, USA). For immunohistochemistry experiments, anti-alpha smooth-muscle actin (αSMA) antibody (ab32575), anti-collagen 1 antibody (ab21286), anti-CD3 antibody (ab16669), anti-F4/80 antibody (ab111101) and rabbit polyclonal IgG (ab27472) were purchased from Abcam (Cambridge, UK), and anti-myeloperoxidase antibody (PA5-16672) purchased from Thermo Fisher Scientific (Waltham, MA, USA). A rabbit ABC Staining System (sc-2018) was purchased from Santa Cruz Biotechnology (Dallas, TX, USA).

### BLM administration

BLM was dissolved in phosphate-buffered saline (PBS). BLM or PBS was administered with osmotic minipumps according to previous reports with minor modifications [19–21]. Briefly, osmotic minipumps (Alzet2001; Durect, Cupertino, CA, USA) containing 200 μl of BLM (125 mg/kg) or PBS were implanted subcutaneously onto the back of WT or ET_B_KO mice aged 6–10 weeks; this was counted as day 0. The pumps delivered 1.0 μg/h for 7 days. The mice were sacrificed on day 28, and skin and lung tissues were taken. Skin samples were taken a short distance from the osmotic minipump to obtain samples that were not affected by pump implantation.

### Histology and immunohistochemistry

The skin and lung samples were fixed in 4 % paraformaldehyde, embedded in paraffin, sectioned, and stained with Masson’s trichrome. To evaluate the BLM-induced scleroderma, we assessed dermal thickening and subcutaneous fat atrophy by measuring the distances between the epidermis and dermis and between the dermis and subcutaneous fat at 40× magnification. The degree of lung fibrosis was quantified using the Ashcroft score at 40× magnification [22]. Collagen 1 deposition area in the dermis was measured with ImageJ software (National Institutes of Health). Fibroblast activation was assessed by αSMA immunohistochemical staining of the skin sections, and αSMA-positive myofibroblasts in the dermis were counted at 100× magnification. To examine whether ET_B_ receptor is involved in the inflammation of this model, we counted myeloperoxidase-positive neutrophils, CD3-positive T lymphocytes and F4/80-positive macrophages in the dermis at 100× magnification.

### Collection of bronchoalveolar lavage fluid (BALF)

To evaluate lung inflammation, a cell count of BALF was performed. Immediately after the mice were sacrificed, a 20-gauge intravenous catheter was inserted into the trachea. A total of 0.5 ml of PBS was instilled three times and withdrawn from the lung via an intratracheal cannula. After BALF was centrifuged, the pellet was resuspended in 1 ml of PBS. Total BALF cells were counted with a hemocytometer. The BALF solutions were stained with May-Giemsa staining after cytospin centrifuge, and the white blood cell differentiation was evaluated by morphological criteria using a light microscope.

### Murine dermal fibroblast isolation and culture

Dermal fibroblasts were isolated from WT and ET_B_KO mice at 3–4 weeks of age according to previous reports, with minor modification [23]. Skin samples were placed dermis-down in culture dishes containing RPMI + 3.6 % dispase, and incubated overnight at 4 °C. The dermis was separated from the epidermis, placed in RPMI + 0.05 % type I collagenase, and incubated at 37 °C for 30 min. The cell suspension was filtered through sterile gauze into a 50-mL conical tube. After adding an equal volume of DMEM + 10 % FBS containing penicillin, streptomycin, and L-glutamine, the suspension was centrifuged at 200 × g for 10 min. Fibroblasts were resuspended in DMEM + 10 % FBS, plated in 100-mm culture dishes, and incubated at 37 °C in 5 % CO_2_ and 95 % room air. For in vitro experiments, fibroblasts were seeded into 6-well plates (1 × 10^5^ per well) and incubated in DMEM + 10 % FBS at 37 °C in 5 % CO_2_. When the fibroblasts had grown to approximately 80 % confluence, the medium was changed and the fibroblasts were treated with 20 μg/ml BLM or 500 nM ET-1 for 24 h. The BLM was dissolved in DMEM without FBS; the ET-1 was dissolved in 0.1 % acetic acid + 0.01 % BSA.

### Quantitative real-time polymerase chain reaction (PCR)

Total RNA was isolated from fibroblasts using an RNeasy Mini kit (Qiagen, Hilden, Germany), followed by cDNA synthesis using a QuantiTect Reverse Transcription kit (Qiagen). PCR reaction mixtures were prepared using the QuantiTect SYBR Green PCR kit (Qiagen), followed by quantitative PCR on a PikoReal system (Thermo Fisher Scientific). The following primer pairs were used: *collagen 1α1* (*Col1α1*), 5’-TGACTGGAAGAGCGGAGAGTACT-3’ (forward) and 5’-GGTCTGACCTGTCTCCATGTTG-3’ (reverse); *αSMA*, 5’-AGAGACTCTCTTCCAGCCATC-3’ (forward) and 5’-GACGTTGTTAGCATAGAGATC-3’ (reverse); *ET-1*, 5’-GTGTCTACTTCTGCCACCTGGACAT-3’ (forward) and 5’-GGGCTCGCACTATATAAGGGATGAC-3’ (reverse); *endothelin receptor type A* (*EDNRA*), 5’-CCTTATCTSCGTGGTCATTG-3’ (forward) and 5’-ACTGAGAGCACAGAGGTTC-3’ (reverse); *endothelin receptor type B* (*EDNRB*), 5’-GGAATCACAGTGCTGAGTC-3’ (forward) and 5’-CTTTGTAGTCCGACGTAATC-3’ (reverse); *glyceraldahyde-3-phosphate dehydrogenase* (*GAPDH*), 5’-AACTTTGGCATTGTGGAAGG-3’ (forward) and 5’-ACACATTGGGGGTAGGAACA-3’ (reverse). *GAPDH* was used as an internal control to normalize the amount of loaded , complementary DNA (cDNA).

### Measurement of soluble collagen content

Sircol collagen assay (Biocolor Ltd., Belfast, Northern Ireland) was used to quantify soluble collagen contents in fibroblast culture supernatant according to the manufacturer’s instructions with minor modification. Briefly, 200 μl of supernatant was mixed with 1 ml of Sircol dye reagent for 30 minutes. After centrifugation, the pellets were dissolved in 1 ml Sircol alkali reagent and vortexed. Relative absorbance was measured at 540 nm.

### Statistical analysis

Data are presented as mean ± standard error of the mean (SEM). Differences between groups were analyzed by Student’s *t* test using GraphPad Prism 5 software (GraphPad Software Inc., La Jolla, CA, USA) and *p* < 0.05 was considered statistically significant.

## Results

### ET_B_KO mice resisted BLM-induced scleroderma

**Fig. 1:**
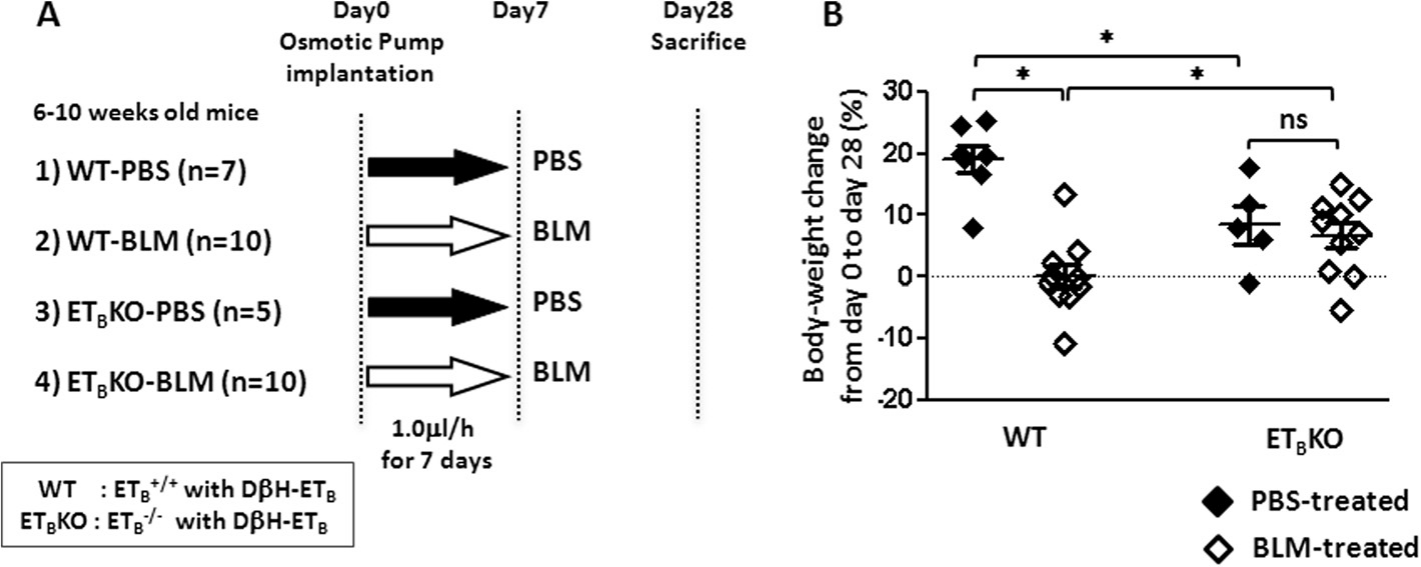
Study design and body-weight changes of each mice group after BLM treatment. **a** Summary of this experimental model and sample number of each group. Osmotic pumps containing 200 μl of BLM (125 mg/kg) or PBS were implanted subcutaneously onto the back of WT or ET_B_KO mice on day 0. Pumps deliver their contents 1.0 μg/h for 7 days. Mice were sacrificed on day28. **b** Body-weight changes from day 0 to day 28 in WT and ET_B_KO mice. The body-weight change was calculated as [(body weight on day 28) − (body weight on day 0)] × (body weight on day 0)^-1^ × 100 (%). Each dot indicates the body-weight change in an individual mouse (^*^ *p* < 0.05). *BLM* bleomycin, *DβH-ET*_*B*_ endothelin type B receptor transgene driven by the human dopamine β-hydroxylase gene promoter, *ET*_*B*_ *KO* endothelin type B receptor knockout, *PBS* phosphate-buffered saline, *WT* wild-type

WT and ET_B_KO mice were treated with BLM or PBS by osmotic minipump. No mice died due to the osmotic minipump implantation or BLM administration. To examine the biological significance of ET_B_ receptor signaling after BLM administration, we measured body-weight changes from day 0 to day 28. ET_B_KO mice were smaller than WT mice of the same age, and ET_B_KO mice gained less body weight than did WT mice when treated with PBS. However, while BLM-treated WT mice did not gain body weight, BLM-treated ET_B_KO mice gained weight similarly to the PBS-treated ET_B_KO mice (Fig 1b).

**Fig. 2:**
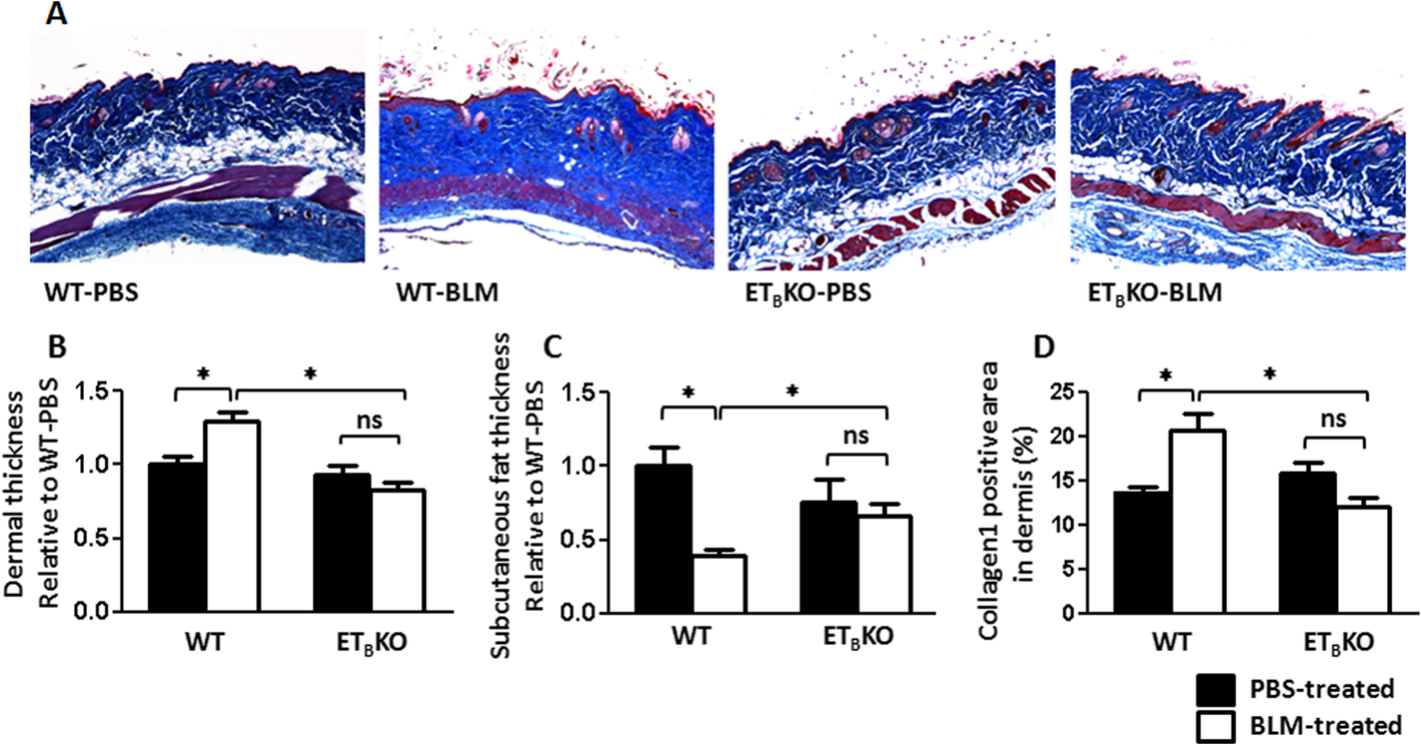
ET_B_KO mice resist BLM-induced skin sclerosis. **a** Representative images of dermis sections stained with Masson's trichrome at 40× magnification. **b** Changes in dermal thickness (epidermal–dermal distance) and **c** subcutaneous fat atrophy (dermal–subcutaneous fat distance) in BLM- or PBS-treated WT and ET_B_KO mice; values are shown as the mean fold change from PBS-treated WT (WT-PBS) mice. **d** Collagen 1 deposition area in dermis of each mice group. (^*^ *p* < 0.05) (n = 5–10 mice per group). *BLM* bleomycin, *ET*_*B*_ *KO* endothelin type B receptor knockout, *PBS* phosphate-buffered saline, *WT* wild-type

To determine the ET_B_ receptor’s role in BLM-induced scleroderma, skin specimens were obtained from each group on day 28 after implanting the osmotic minipump. The skin samples were stained with Masson’s trichrome to evaluate the dermal thickness and subcutaneous fat atrophy. In WT mice, BLM treatment increased the distance between the epidermis and dermis, and reduced the distance between the dermis and subcutaneous fat. In contrast, these distances did not change significantly in the ET_B_KO mice treated with PBS or BLM (Fig. 2a-c). Likewise, collagen 1 deposition area in the dermis was increased by BLM-treatment in WT mice, but the increment was not seen in ET_B_KO mice (Fig. 2d). These results suggested that ET_B_ receptor signaling is associated with BLM-induced skin sclerosis. Also lung fibrosis and inflammation were evaluated, but neither cell counts in BALF nor lung histological scores were not significantly different between WT and ET_B_KO with BLM treatment (Additional file 1: Figure S1).

### Inhibited fibroblast activation protects ET_B_KO mice against BLM-induced scleroderma

BLM-induced scleroderma is associated with the differentiation of fibroblasts into myofibroblasts. These myofibroblasts, which are identified by αSMA expression, promote fibrosis by producing collagen and other extracellular matrix components [24, 25].

**Fig. 3:**
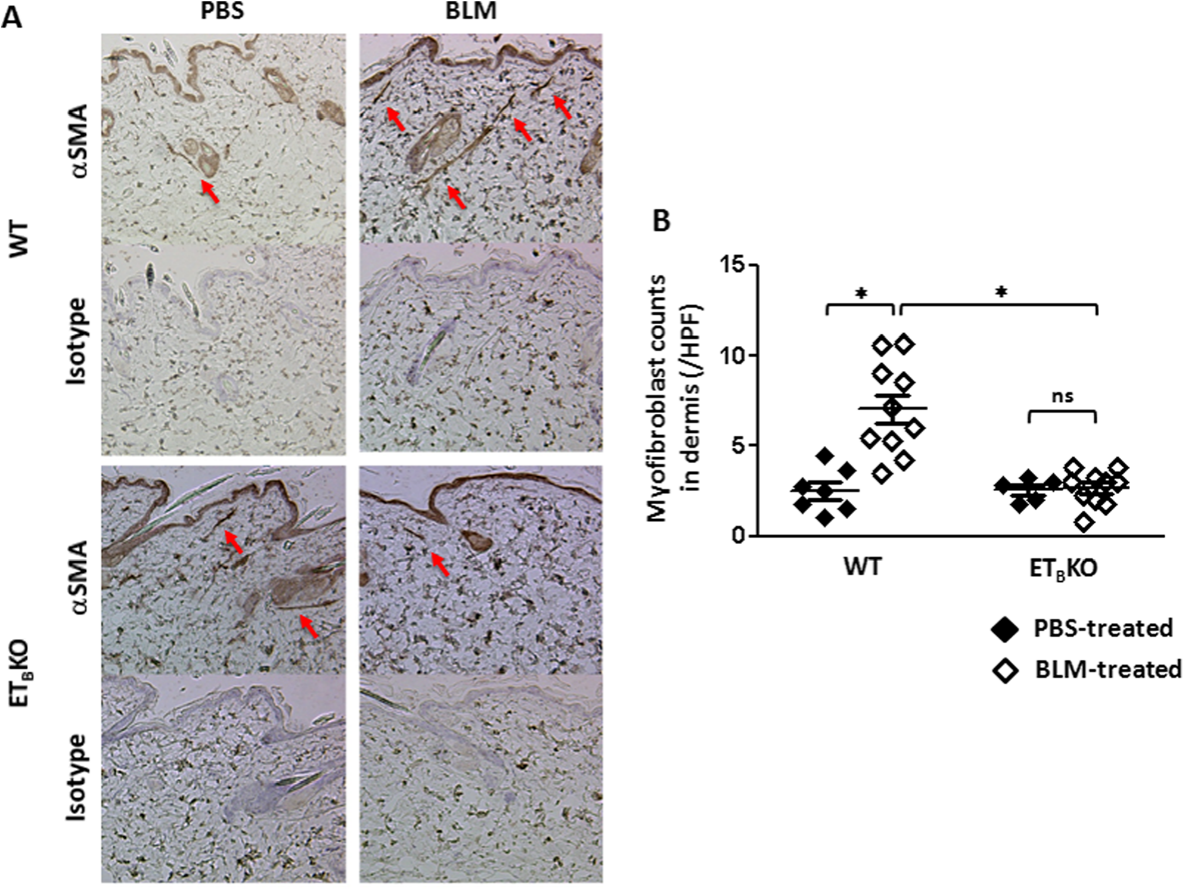
Fewer αSMA-expressing myofibroblasts are observed in the dermis of ET_B_KO than WT mice after BLM treatment. **a** Representative images showing the immunohistochemical staining of skin samples for αSMA (*upper*) and isotype (*lower*) at 100× magnification. *Red arrows* indicate myofibroblasts (αSMA-expressing spindle-shaped cells). **b** Average myofibroblast counts per field of view in the dermis, counted at 100× magnification (^*^ *p* < 0.05); n = 5–10 mice per group*. BLM* bleomycin, *HPF* high-power field, *ET*_*B*_ *KO* endothelin type B receptor knockout, *PBS* phosphate-buffered saline, *WT* wild-type

To determine whether ET_B_ receptor signaling contributes to BLM-induced fibroblast differentiation, we counted the number of αSMA-positive cells in the dermis of BLM- or PBS-treated WT and ET_B_KO mice. BLM increased the number of αSMA-positive myofibroblasts in the WT but not ET_B_KO dermis, indicating that ET_B_ is involved in myofibroblast formation (Fig. 3).

**Fig. 4:**
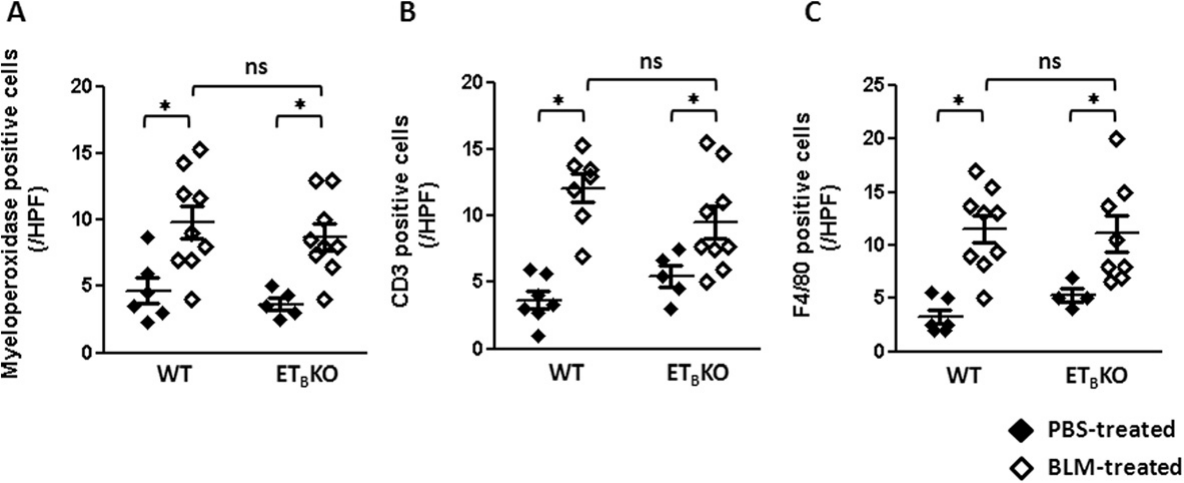
Infiltration of inflammatory cells in the dermis of WT and ET_B_KO mice after BLM treatment. The average cell counts of **a** myeloperoxidase, **b** CD3, and **c** F4/80-positive cells in the dermis. The cells were counted per field of view at 100× magnification; n = 5–10 mice per group. (^*^ *p* < 0.05). *BLM* bleomycin, *ET*_*B*_ *KO* endothelin type B receptor knockout, *HPF* high-power field, *PBS* phosphate-buffered saline, WT wild-type

Inflammatory cell filtration in the dermis was counted to determine whether the degree of inflammation was different between WT and ET_B_KO skin fibrosis. The numbers of myeloperoxidase-positive neutrophils, CD3-positive T cells and F4/80-positive macrophages in the dermis were significantly increased when treated with BLM. However, the numbers of these inflammatory cells were not different between WT and ET_B_KO mice both before and after BLM treatment (Fig. 4).

Collectively ET_B_ receptor signaling seemed to contribute to fibroblast activation independent of inflammation.

### The ET_B_ receptor is associated with dermal fibroblast activation and collagen synthesis in response to BLM or ET-1

**Fig. 5:**
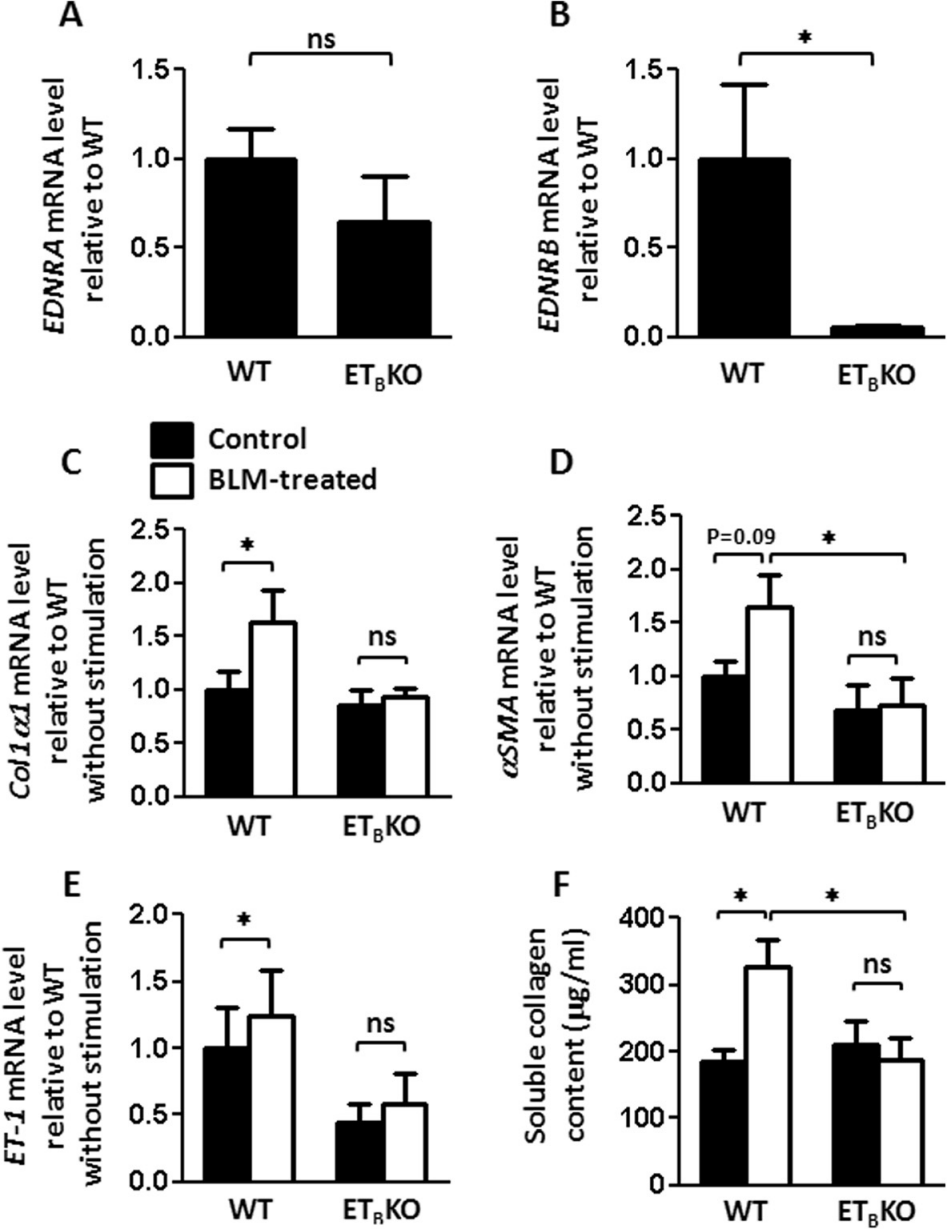
Knockout of the ET_B_ receptor signal reduces the activation of skin-derived fibroblasts in response to BLM. **a** *EDNRA* and **b** *EDNRB* mRNA levels in skin-derived fibroblasts from WT and ET_B_KO mice (n = 5 each). **c**-**e** BLM-induced fibroblast activation in WT and ET_B_KO mice (n = 6–9 each), determined by the gene expression levels of *Col1α1*, *αSMA,* and *ET-1*. Values show the mean mRNA levels relative to WT without stimulation (^*^ *p* < 0.05). **f** Soluble collagen production from WT and ET_B_KO (n = 4 each) fibroblasts with or without BLM treatment (^*^ *p* < 0.05). *WT* wild-type, αSMA α-smooth muscle actin, *BLM* bleomycin, *Col1α1* collagen 1α1, *ET-1* endothelin-1, *ET*_*B*_ *KO* endothelin type B receptor knockout, *PBS* phosphate-buffered saline

Because fibroblasts play a critical role in fibrosis, we next examined the characteristics of dermal fibroblasts from WT and ET_B_KO mice. ET-1 or BLM stimulation is reported to induce fibroblasts to express profibrotic genes [12, 26]. Thus, we isolated dermal fibroblasts from WT and ET_B_KO mice and assessed their activation and collagen synthesis in response to ET-1 or BLM in vitro. Dermal fibroblasts from ET_B_KO mice expressed little or no *EDNRB* mRNA but expressed *EDNRA* normally, as expected (Fig. 5a, b).

BLM stimulation induced *Col1α1* and *ET-1* gene expression in fibroblasts from WT mice, but not in those from ET_B_KO mice. When stimulated with BLM, WT fibroblasts expressed the *αSMA* gene at significantly higher levels than did ET_B_KO fibroblasts (Fig. 5c-e).

**Fig. 6:**
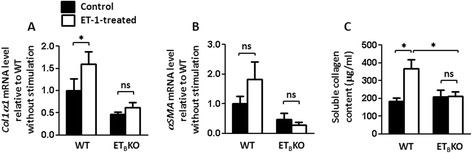
Knockout of the ET_B_ receptor signal reduces the activation of skin-derived fibroblasts in response to ET-1. **a, b** ET-1-induced fibroblast activation in WT and ET_B_KO mice (n = 4 or 5 each), determined by the gene expression levels of *Col1α1*, *αSMA.* Values show the mean mRNA levels relative to WT without stimulation (^*^ *p* < 0.05). **c** Soluble collagen production from WT and ET_B_KO (n = 4 each) fibroblasts with or without ET-1-treatment (^*^ *p* < 0.05). *αSMA* α-smooth muscle actin, *BLM* bleomycin, *Col1α1* collagen 1α1, *ET-1* endothelin-1, *ET*_*B*_ *KO* endothelin type B receptor knockout, *PBS* phosphate-buffered saline, *WT* wild-type

ET-1 stimulation induced *Col1α1* expression in WT fibroblasts. ET-1 also tended to induce *αSMA* mRNA in WT fibroblasts, although this increase did not reach statistical significance. ET-1 stimulation did not influence the *Col1α1* or *αSMA* expression in ET_B_KO fibroblasts (Fig. 6a, b).

Next, we measured soluble collagen content in the supernatant with Sircol collagen assay to assess whether BLM or ET-1 stimulation increases collagen production by fibroblasts. BLM or ET-1 treatment of WT fibroblasts significantly induced collagens. Increase in collagen production by BLM or ET-1 stimulation was not seen in ET_B_KO fibroblasts (Figs. 5f and 6c). Collectively, ET_B_KO fibroblasts showed less profibrotic properties in response to BLM or ET-1 than WT.

## Discussion

In this study, we demonstrated for the first time with genetic ET_B_KO mice that ET_B_ receptor signaling in the presence of the ET_A_ receptor promotes fibrosis in BLM-induced scleroderma. Myofibroblast formation was inhibited in ET_B_KO mice, allowing them to resist BLM-induced skin fibrosis. At least one mechanism of the ET_B_ receptor appears to function through dermal fibroblasts, because the ET_B_KO fibroblasts did not respond to BLM or ET-1 stimulation, whereas WT fibroblasts showed increased *Col1α1* and *αSMA* expression in vitro.

Rescued ET_B_KO rats and mice, like those used in this study, are useful for investigating ET_B_ receptor signaling. ET_B_KO rats show deteriorating health due to hypoxia-induced pulmonary hypertension, and ET_B_KO mice show worsened vascular remodeling in a carotid artery ligation model [27, 28]. Our study is the first to demonstrate that the ET_B_ receptor is critical for BLM-induced scleroderma using ET_B_KO mice.

ET-1 is important in fibrosis because it induces collagen production and myofibroblasts. We found that in WT fibroblasts, BLM induced ET-1, which in turn induced collagen production. On the other hand, ET_B_KO fibroblasts did not produce collagen when stimulated by ET-1, indicating that ET_B_ is indispensable for the ET-1-induced collagen production. Thus, the ET-1–ET_B_ system is critical for the collagen production by fibroblasts.

We showed that myofibroblast formation was strongly inhibited in the ET_B_KO mice in vivo. In addition, ET_B_KO fibroblasts did not express *αSMA* mRNA in response to BLM or ET-1. These results indicate that ET_B_ is involved in myofibroblast formation. In our experiments, ET-1’s induction of *αSMA* in WT fibroblasts in vivo was stronger than that in vitro. This was not surprising, because there are multiple sources of myofibroblasts in vivo, including tissue-resident fibroblasts [29], epithelial or endothelial cells transitioning into mesenchymal cells (EMT or EndoMT) [30–33], or bone marrow-derived circulating fibrocytes [34, 35]. Thus, we believe that ET_B_ is involved in myofibroblast formation from various types of cells.

Myofibroblast precursors differ in various tissues. For example, resident fibroblasts are the main contributors to myofibroblasts in lung fibrosis [36], and hepatic stellate cells are the most important source of myofibroblasts in liver fibrosis [37]. Although a recent study found that adipocytes differentiate into myofibroblasts in dermal fibrosis [38], the primary source of myofibroblasts in dermal fibrosis remains unclear. ET_B_ receptor signaling seems to be important in liver and cardiac fibrosis [39–41], but not renal fibrosis [42]. The difference of the skin and lung fibrosis might come from the difference in both myofibroblast precursor and contribution of ET_B_ receptor signaling in the organ. Further investigation is needed to reveal which cells are responsible for generating dermal myofibroblasts, and how the ET_A_ and ET_B_ receptors are involved in dermal or other organ fibrogenesis.

In dermal fibroblasts, both ET_A_ and ET_B_ receptor signaling are reported to be necessary for ET-1 to exert its profibrotic effect [12]; in that study, neither ET_A_ nor ET_B_ receptor selective antagonists inhibited the collagen synthesis in dermal fibroblasts stimulated with ET-1. Our data appear to conflict with that study; this discrepancy might be due to differences between a pharmacological blockade and genetic knockout. The genetic knockout is assumed to affect permanently and completely, while the effect of pharmacological blockade is usually temporary and depends on affinity. We did not perform in vivo and in vitro study with the ET_B_ selective antagonist, because it was reported that existing inhibitors could not suppress the fibroblast activation in vitro as mentioned above. We hope that new ET_B_ selective antagonists that suppress fibroblast function will be developed.

Clinically, dual ET_A_/ET_B_ receptor antagonists (bosentan, macitentan) and a selective ET_A_ receptor antagonist (ambrisentan) improve the hemodynamics, exercise capacity, and survival rate in patients with pulmonary arterial hypertension [9–11]. These ET receptor blockers are also effective for preventing and improving digital ulcers in SSc patients [43, 44]. Currently, there is no selective ET_B_ receptor antagonist that can be safely administered to humans. However, our study suggests that ET_B_ receptor blockade is a potential pharmaceutical treatment for progressive skin fibrosis in SSc patients.

## Conclusions

This study described that ET_B_KO mice were resistant to BLM-induced skin sclerosis and that ET_B_KO mice showed less fibroblast activation and myofibroblast formation in response to BLM or ET-1. Thus, ET-1–ET_B_ receptor signaling is involved in skin sclerosis and in collagen synthesis by dermal fibroblasts.

## Additional file

Additional file 1:
**Figure S1.** BLM-induced lung inflammation and fibrosis of WT and ET_B_KO mice. (A) Total cell, (B) macrophages, (C) neutrophils, and (D) lymphocytes counts in BALF collected from WT and ET_B_KO with PBS or BLM treatment (n = 5–10 mice per group) on day28 after osmotic implantation (^*^ *p* < 0.05). (E) Representative images of lung sections stained with Masson's trichrome at 40 × magnification. (F) Ashcroft score of WT and ET_B_KO mice with PBS or BLM treatment (n = 5–10 mice per group) (^*^ *p* < 0.05). *BLM* bleomycin, *ET*_*B*_ *KO* endothelin type B receptor knockout, *PBS* phosphate-buffered saline, *WT* wild- type. (TIF 1160 kb)

## Abbreviations

αSMA: alpha-smooth muscle actin
BALF: bronchoalveolar fluid lavage
BLM: bleomycin
BSA: bovine serum albumin
cDNA: complementary DNA
Col1α1: collagen 1α1
DMEM: Dulbecco's modified Eagle's medium
DβH: human dopamine β-hydroxylase
EDNRA: endothelin receptor type A
EDNRB: endothelin receptor type B
ET-1: endothelin-1
ET_A_: endothelin type A
ET_B_: endothelin type B
ET_B_KO: endothelin type B receptor-knockout
FBS: fetal bovine serum
GAPDH: glyceraldahyde-3-phosphate dehydrogenase
PBS: phosphate-buffered saline
PCR: polymerase chain reaction
SEM: standard error of the mean
SSc: systemic sclerosis
WT: wild-type
