# Supplementary figures and images for: Knockout of endothelin type B receptor signaling attenuates bleomycin-induced skin sclerosis in mice
Source: Arthritis Res Ther. 2016 May 21;18:113. doi: 10.1186/s13075-016-1011-4 (PMC4875589; doi:10.1186/s13075-016-1011-4)

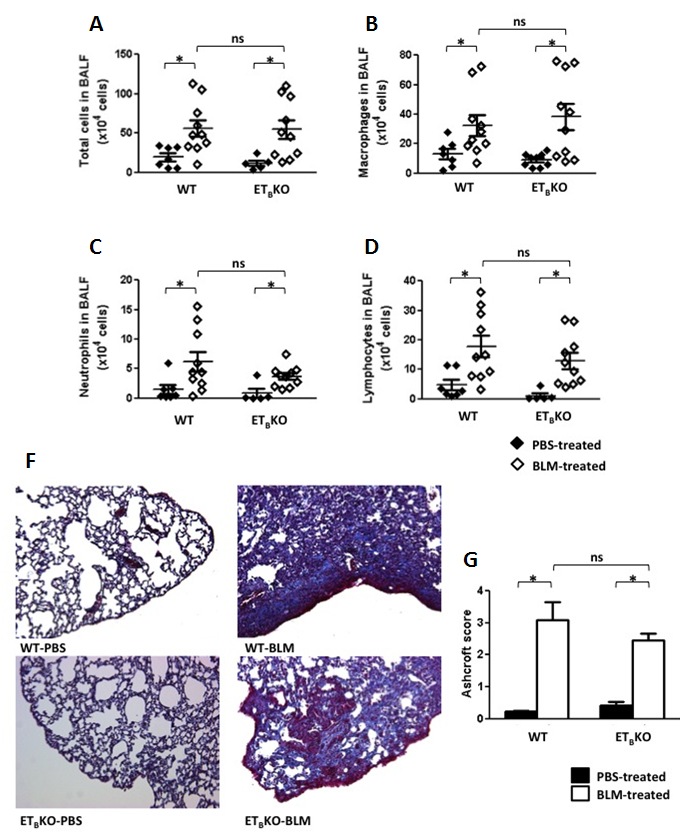

Supplement: Additional file 1: — Figure S1. BLM-induced lung inflammation and fibrosis of WT and ETBKO mice. (A) Total cell, (B) macrophages, (C) neutrophils, and (D) lymphocytes counts in BALF collected from WT and ETBKO with PBS or BLM treatment (n = 5–10 mice per group) on day28 after osmotic implantation (* p < 0.05). (E) Representative images of lung sections stained with Masson's trichrome at 40 × magnification. (F) Ashcroft score of WT and ETBKO mice with PBS or BLM treatment (n = 5–10 mice per group) (* p < 0.05). BLM bleomycin, ET B KO endothelin type B receptor knockout, PBS phosphate-buffered saline, WT wild- type. (TIF 1160 kb) [file 13075_2016_1011_MOESM1_ESM.tif]
